# Automated assay for screening the enzymatic release of reducing sugars from micronized biomass

**DOI:** 10.1186/1475-2859-9-58

**Published:** 2010-07-16

**Authors:** David Navarro, Marie Couturier, Gabriela Ghizzi Damasceno da Silva, Jean-Guy Berrin, Xavier Rouau, Marcel Asther, Christophe Bignon

**Affiliations:** 1 UMR 1163 INRA/Universités de Provence et de la Méditerranée, Unité de Biotechnologie des Champignons Filamenteux, IFR86-BAIM, ESIL, 163 avenue de Luminy CP 925, 13288 Marseille cedex 09, France; 2 Architecture et Fonction des Macromolécules Biologiques, UMR 6098, CNRS et université d'Aix-Marseille I et II, 163 avenue de Luminy CP 925, 13288 Marseille cedex 09, France; 3 UMR 1208 INRA/SupAgro/CIRAD/Université Montpellier 2, Unité Ingéniérie des Agropolymères et Technologies Emergentes, 2 place Pierre Viala, 34060 Montpellier cedex 01 France

## Abstract

**Background:**

To reduce the production cost of bioethanol obtained from fermentation of the sugars provided by degradation of lignocellulosic biomass (*i.e*., second generation bioethanol), it is necessary to screen for new enzymes endowed with more efficient biomass degrading properties. This demands the set-up of high-throughput screening methods. Several methods have been devised all using microplates in the industrial SBS format. Although this size reduction and standardization has greatly improved the screening process, the published methods comprise one or more manual steps that seriously decrease throughput. Therefore, we worked to devise a screening method devoid of any manual steps.

**Results:**

We describe a fully automated assay for measuring the amount of reducing sugars released by biomass-degrading enzymes from wheat-straw and spruce. The method comprises two independent and automated steps. The first step is the making of "substrate plates". It consists of filling 96-well microplates with slurry suspensions of micronized substrate which are then stored frozen until use. The second step is an enzymatic activity assay. After thawing, the substrate plates are supplemented by the robot with cell-wall degrading enzymes where necessary, and the whole process from addition of enzymes to quantification of released sugars is autonomously performed by the robot. We describe how critical parameters (amount of substrate, amount of enzyme, incubation duration and temperature) were selected to fit with our specific use. The ability of this automated small-scale assay to discriminate among different enzymatic activities was validated using a set of commercial enzymes.

**Conclusions:**

Using an automatic microplate sealer solved three main problems generally encountered during the set-up of methods for measuring the sugar-releasing activity of plant cell wall-degrading enzymes: throughput, automation, and evaporation losses. In its present set-up, the robot can autonomously process 120 triplicate wheat-straw samples per day. This throughput can be doubled if the incubation time is reduced from 24 h to 4 h (for initial rates measurements, for instance). This method can potentially be used with any insoluble substrate that is micronizable. A video illustrating the method can be seen at the following URL: http://www.youtube.com/watch?v=NFg6TxjuMWU

## Background

Bioethanol is the major surrogate for liquid fossil fuels. The production of second generation bioethanol requires two consecutive steps [1]: monomeric sugars are first released from lignocellulosic biomass, and then fermented into ethanol by a suitable microbe such as the yeast *Saccharomyces cerevisiae *[2].

The releasing of fermentable sugars from lignocellulosic biomass is also a two-step process. In the first step, the cellulose embedded within a matrix of hemicellulose, pectin and lignin is made more accessible using physico-chemical pre-treatments. In the second step, the accessible cellulose is degraded into oligo/monomeric glucose by the action of biomass-degrading enzymes typically secreted by filamentous fungi [3].

One of the reasons why second-generation bioethanol carries higher production costs than petroleum-derived gasoline is that fungal-based hydrolytic enzymes are expensive to produce. Therefore, major efforts are now focused on lowering enzyme-related costs in cellulosic biorefineries [4].

At industrial level, *Trichoderma *and *Aspergillus *are the most-widely used filamentous fungi for producing biomass-degrading enzyme-containing secretomes [4], although the genome of *T. reesei *QM6a strain carries few genes likely to encode for the enzymes involved in biomass degradation [5]. This strain has undergone several rounds of mutation/selection to increase its capacity to produce and secrete cellulases at high yields. As a result, the industrial strain *T. reesei *CL847 secretes as much as 30 g of proteins per liter of culture medium, and proteomic analysis of this secretome reveals that most of the proteins identified by mass spectrometry are biomass-degrading enzymes [6]. This extensive selection process means that the capacity of CL847 to produce and secrete cellulolytic activities could soon reach an impassable limit. Therefore, other means for reducing enzyme costs must now be considered.

One option is to reduce the amount of enzymes used by increasing the overall specific activity of the enzymatic cocktails. This can be achieved by searching for enzymatic activities which could complement those of the already improved *T. reesei *strain CL847 secretome.

Finding such complementing activities from within natural biodiversity entails screening huge numbers of samples. Using a robot under sterile conditions, we recently set-up a miniaturized fungal culture method in 16-well plates [7] that is currently used to grow wild filamentous fungi. We reasoned that coupling small-scale fungal cultures with automated analysis of the sugar-releasing activity of their secretomes within a single robot would create a powerful tool for screening at high-throughput for new biomass-degrading activities.

Manual [8,9] and semi-automated [10] microplate-based methods using artificial substrates have been described already, and the new generation of manual [11] and partially automated [12] microplate methods can use micronized biomass to assess these enzymatic activities. Several of these methods make use of 3,5-dinitrosalicylic acid (DNS) [8,10,11] to assay the reducing sugars released by the enzymes because DNS assay is particularly suited to the microplate format.

The use of microplates for performing enzymatic digestion and reducing sugar assays using DNS were real technological breakthrough. Unfortunately, although some steps were automated the published methods still included one or more manual steps hampering throughput.

Therefore, using tools implemented by these previous set-ups, we fully automated a small-scale assay for screening the sugar-releasing activity of biomass-degrading enzymes on natural substrates such as wheat-straw and spruce.

## Methods

### Micronized substrates

The starting raw material consisted of dry minced wheat-straw (~0.5-1 cm *Triticum aestivum *cv Apache, France, 2006) and spruce (~1-5 cm chips, Holmen plant, Braviken Sweden, 2006).

Substrate powders were prepared using successive grinding steps, as described below.

a) Wheat-straw and spruce were ground using a Retsch SM2000 cutting mill with a 2 mm square mesh.

b) Wheat-straw was further comminuted using 4 consecutive 12,000 rpm centrifugal millings and 4 meshes with decreasing trapezoid hole size (1, 0.5, 0.25, 0.12 mm) in a Retsch ZM200 ultra-centrifugal mill. At the end of the micronization process, the whole wheat-straw powder was used.

c) Spruce was comminuted using a single 18,000 rpm centrifugal impact mill in a type Hosokawa-Alpine model 100 UPZ station with a 0.3 mm selection screen.

Additional file 1 provides pictures of the substrates at different stages of size reduction.

Powder particle size was measured at room temperature using a Coulter LS230 laser diffraction granulometer. The dispersion index (di) was determined as (d_90_-d_10_)/d_50_, where d_90_, d_50 _and d_10 _represent the particle size below which 90%, 50% and 10% particles are found, respectively [13]. Each sample was measured in duplicate.

The carbohydrate content of micronized substrates was determined by gas-liquid chromatography (GLC). After hydrolysis (30 min at 25°C in presence of 36N H_2_SO_4_, followed by 2 h at 100°C in presence of 2N H_2_SO_4_) and alditol acetate derivatization [14], alditol acetates were resolved by GLC on a DB225 capillary column (J&W Scientific) with allose as chromatography standard. Glucan content was calculated as the sum of anhydro-glucose content, and hemicellulose content was calculated as the sum of anhydro-arabinose, anhydro-xylose, anhydro-mannose and anhydro-galactose contents.

It is not possible to determine the carbohydrate content of minced substrate by this method because the acid-hydrolysis step requires smaller size particles. Therefore, the carbohydrate content of non-micronized wheat-straw was performed on wheat-straw ground using a Cyclotec (Tecator) and a 0.3 mm mesh.

### Substrate-containing plates

Substrate suspensions were made by adding 1 g of micronized wheat-straw or 2 g of micronized spruce to 100 mL of 50 mM sodium acetate pH5. To prevent any contamination, 30 μg/mL cycloheximide, and 40 μg/mL tetracycline were added [9,12]. Suspensions were allowed to hydrate overnight at 4°C without stirring.

To fill the plates, 3 × 100 μL of suspension was aspirated by the robot using 3 pre-cut 250 μL carbon tips, and then the whole volume contained in one tip (100 μL) was loaded into each of 66 wells of a flat-bottom polypropylene 96-well plate (Costar reference 3364, Corning Life Sciences (USA)) following the dispensing pattern described in Additional file 2A. During aspiration, the suspension (100 mL in a 250 mL beaker) was continuously shaken using a magnetic stirrer. Once all wells were filled, the robot heat-sealed the plate, which was then stored frozen at -20°C until use. Ten plates (~66 mL of substrate suspension) were prepared per hour.

Plate-to-plate filling reproducibility was checked by weighting plates before and after filling (Additional file 2B and 2C).

### Enzymatic release of sugars from biomass

After thawing, a glucose reference scale was added to the substrate-containing plates just before use: 8 × 1 mL tubes were placed on the robot bench, each containing one of the 8 glucose dilutions in 50 mM sodium acetate pH5. The robot dispensed 125 μL of each dilution into wells A1 (0 mM) to H1 (20 mM) (Additional file 2A). Wells in lines A and E (columns 2 to 12) were filled with buffer without substrate and were used as substrate-free negative controls.

Enzymes (25 μL) were then added to the scheduled wells. See Additional file 3 for a detailed description of enzymes and providers. *T. reesei *CL847 secretome was used a reference cellulolytic activity [15] and is referred to as "E508" throughout the text.

When enzymatic activities were expressed as percentages, 100% was E508 activity at 1/50 dilution (E/50, *i.e*. 30 μg per well). The enzymes were used at the following dilutions: E/50, E508 (1/50 dilution); E/200, E508 (1/200 dilution); D6/100, Depol 686 L (1/100 dilution); D7/100, Depol 740 L (1/100 dilution); H/10, Hemicellulase (1/10 dilution); X/10, Xylanase (1/10 dilution); P/20, Pectinex Ultra SPL (1/20 dilution); N/100, Novozyme 188 (1/100 dilution); V/100, Viscozyme L (1/100 dilution); C/20, Celluclast 1.5 L (1/20 dilution); F/20, Fungamyl 800 L (1/20 dilution).

**Figure 1:**
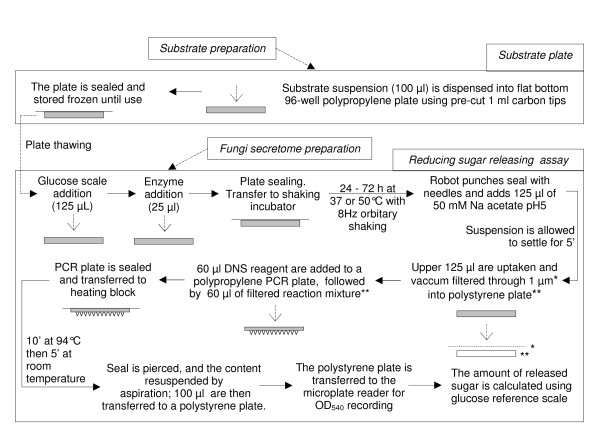
**Synopsis of the assay**. Indicated volumes are for one well. Upper panel, automated substrate plate preparation. Lower panel, automated sugar release assay. Except for freezing/thawing the substrate-containing plate that are performed manually, all steps are performed autonomously by the robot.

The plate was then heat-sealed and transferred to the shaking incubator. Enzymatic release of sugar was allowed to proceed with constant shaking (8 Hz) at 37°C or 50°C for different lengths of time, and the plate was then processed as described in Figure 1. At the end of the experiment, the robot computer automatically translated OD_540 _into "glucose equivalent μmol" per well using the glucose scale internal to each plate.

For results expressed as percentages, the average value of a triplicate experiment (glucose equivalent μmol) obtained in the absence of enzymes (negative controls, Additional file 2A) was subtracted from the average of each experimental result. The resulting difference was then expressed as a percentage of reference enzymatic activity (E/50).

Calibration of the sugar-releasing experiment

a) Substrate concentration

The amount of reducing sugars released from different concentrations of micronized substrate suspensions was assayed after 24 h incubation at 37°C in presence of E/50. For each substrate concentration, background was determined by incubating the relevant substrate concentration in the absence of enzyme.

b) Enzyme concentration

The amount of reducing sugars released from 1% (wheat-straw) or 2% (spruce) (w/v) substrate suspension by different E508 concentrations was assayed after 24 h incubation at 37°C. For each enzyme concentration, the "enzyme background" was determined by incubating the relevant enzyme concentrations in the absence of substrate. The "substrate background" (single point) was obtained by incubating 1% (wheat-straw = 0.11 μmol glucose equivalent) or 2% (spruce = 0 μmol glucose equivalent) substrate suspension in the absence of enzyme. For each enzyme concentration, net sugar release was determined by subtracting the two backgrounds from raw data.

c) Incubation time

The amount of reducing sugars released by E/50 from 1% (wheat-straw) or 2% (spruce) substrate suspension was assayed after different incubation durations at 37°C. For each time point, two backgrounds were determined: one was obtained by incubating a constant amount of substrate (1% (wheat-straw) or 2% (spruce) suspension) for the relevant duration in the absence of E/50 the other was obtained by incubating a constant amount of E/50 for the relevant duration in the absence of substrate. For each timepoint, net sugar release was determined by subtracting the two backgrounds from raw data.

### Reducing sugar assay

This assay, based on the 3,5-dinitrosalicylic acid (DNS) method [8,10,11,16], was performed as described in Figure 1. The DNS reagent (5 g DNS and 150 g sodium potassium tartrate dissolved in 0.5 L of 0.4 N sodium hydroxide) was stored in the dark at room temperature. The following 96-well plates were used in the assay:

- PCR plate: twin.tec PCR plate, Eppendorf (USA) reference 951020460, skirted.

- Filtration plate: Acroprep 96, 1 μm A/B Glass, PALL (USA) reference PN 5031.

- Polystyrene plate: flat bottom, sterile, IWAKI (Japan) reference 3861.

### Robot set-up (Figure 2)

**Figure 2:**
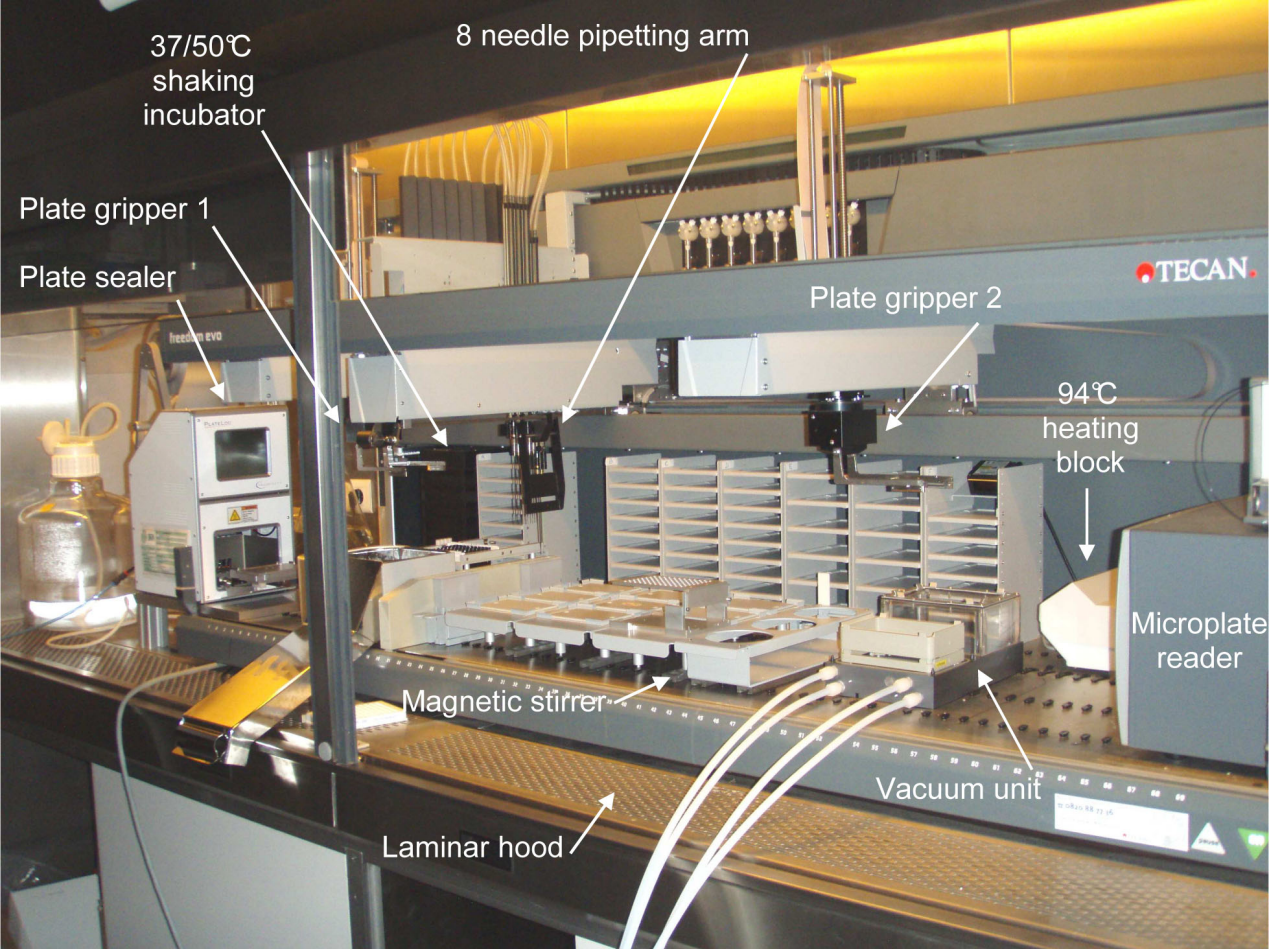
**Robot set-up**. The robot is housed in a laminar hood so that experiments can be performed under sterile airflow. Outside the hood, and not visible on the picture, the air compressor used with the plate-sealer and the computer driving the robot and the allied tools are located on the left and on the right of the hood, respectively. The tools used for biomass sugar-releasing assay are indicated by arrows.

The robot was a GENESIS Freedom Evo (Tecan) comprising an 8 needle pipetting arm (with three needles extemporaneously replaced by pre-cut carbon tips for dispensing substrate suspension; see 'substrate-containing plates' above), 2 handling arms, each with a plate gripper for moving microplates (the left arm was used to transfer plates between plate sealer and shaking incubator, and the right arm was used for all other plate shifts), an Infinite M1000 microplate reader (Tecan), a TE-VACS vacuum unit (Tecan), a THERMOSTAT heating block (Eppendorf) preset at 94°C which could be switched on/off by one robot arm, a shaking incubator preset at 37°C or 50°C (Tecan), and a PlateLoc automatic plate-sealer (Agilent USA) using heat-sealable aluminium foil that could be pierced by the robot needles. The pressure required for sealing plates was provided by a 2xOF302-40B air compressor (JUN-AIR France).

All the tools (except the magnetic stirrer and the heating block temperature-setter) were controlled directly by the robot computer.

Except for manual freezing/thawing of substrate-containing plates, all steps were performed autonomously by the robot.

### Assessment of microplate sealing homogeneity

The 8 concentrations of the glucose reference scale (0, 2, 4, 6, 8, 10, 15, 20 mM, see lane 1 in Additional file 2AA) were loaded into lanes 1 to 12 of a polypropylene plate normally used for making substrate plates so that all 96 microplate wells were filled with the glucose scale only. After filling, the plate underwent the full reducing sugar-release assay described in Figure 1 as if it was a regular substrate plate, except that no enzyme was added. Incubation at 37°C was for 72 h. At the end of the experiment, the DNS data provided by the microplate reader were used to calculate the slope (OD_540_/theoretical glucose concentration (mM)) and the correlation coefficient of each of the 12 glucose reference scales.

## Results and discussion

### Sealing

An automated assay set-up requires an automatic microplate sealer. Preliminary tests using manual and semi-automatic heat-sealers had provided disappointing results: although sealing could be made efficient (no evaporation loss), it required extended heating periods that could have damaged the test enzymes and often modified the microplate shape causing the robot gripper problems with subsequent handling. Conversely, we encountered evaporation losses (uneven sealing) when preserving plate shape and enzymatic activity by lowering the temperature and/or shortening the sealing time (not illustrated).

**Figure 3:**
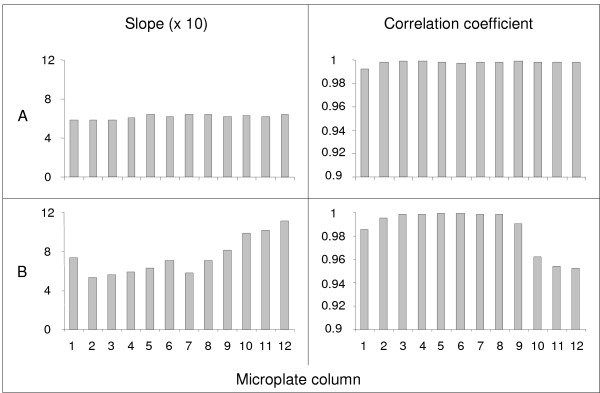
**Automatic heat-sealing (A) *vs *manual sealing using adhesive tape (B)**. The glucose reference scale was processed as described in the Methods section. The figure reports the resulting slope and the correlation coefficient of each of the 12 glucose reference scales loaded in each of the 12 microplate columns.

In addition to allowing the assay to be entirely automated, the automatic sealer happened to solve these two issues: it provided perfectly even sealing without compromising plate shape or enzymatic activity. As an illustration of this efficiency, the data reported in Figure 3 compare the results obtained by automatic heat sealing and by manual sealing using an adhesive tape. Although both sealing protocols used aluminium foil, the 12 reference glucose scales exhibited identical slopes with good correlation coefficients when automatically heat sealed, whereas there were discrepancies due to uneven and deficient sealing when a simple adhesive sheet was used.

### Substrates

**Figure 4:**
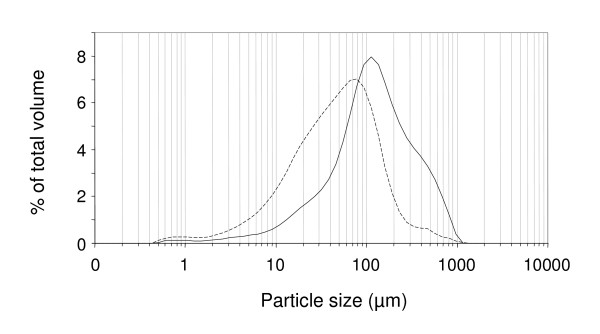
**Particle size distribution of micronized biomass**. Wheat-straw (broken line) and spruce (solid line) particle size distribution from 0 to 10 mm.

To be usable as suspensions in an automated 96-well plate-format assay, the substrates (wheat-straw and spruce) had to be reduced in size to roughly that of flour particles (~100 μm). This goal was achieved by devising the multi-step micronization procedure described in 'Methods'. Although the resulting wheat-straw and spruce powders made suspensions that could be easily pipetted by the robot, the size distribution of their constituent particles did not completely overlap due to different grinding protocols and was characterized by a degree of dispersion (Figure 4, Table 1A). However, this dispersion had no effect on plate-to-plate dispensing reproducibility (Additional file 2BB and 2CC), and even on well-to-well dispensing reproducibility as suggested by the low standard deviations of sugar-releasing assays using these substrate-containing plates (see further).

**Table 1:** Micronized biomass features

A	μm (standard deviation*)										
	**d_10_**				**d_50_**	**d_90_**			**di**		

Wheat-straw	8.3 (1.0)				42.6 (0.0)	128.9 (2.2)			2.8 (0.1)		

Spruce	22.2 (0.4)				106.1 (1.1)	387.6 (2.3)			3.4 (0.0)		

			g/100 g (standard deviation*)								
B	Pore size										
	(mm)		Arabinose		Xylose	Mannose		Galactose		Glucose	

Wheat-straw	0.3		3.9 (0.2)		20.5 (1.1)	< 1		**		36.3 (1.8)	

	0.12		3.9 (0.1)		19.3 (0.9)	< 1		2.0 (0.3)		37.3 (1.0)	

Spruce	0.3		1.4 (0.1)		6.6 (0.0)	13.6 (0.3)		2.4 (0.6)		54.4 (1.3)	

A, Particle size distribution of micronized biomass. Using data from Figure 4, the dispersion index (di) was determined as described in 'Methods'; *of a duplicate experiment. B, the sugar composition of micronized biomass passing through the indicated pore size was analyzed as described in 'Methods'; *of a triplicate experiment; **Not determined.

To evaluate whether micronized substrates were faithful surrogates for the original biomass, we analyzed their carbohydrate composition. Unfortunately, only particles below 0.3 mm could be submitted to acid hydrolysis followed by GLC analysis. Consequently, carbohydrate content could be compared for wheat-straw particles that had been ground through 0.3 and 0.12 mm grids but not for spruce particles that had been prepared using a single 0.3 mm grid. Table 1B indicates that both 0.3 and 0.12 mm wheat-straw fractions had comparable carbohydrate compositions, suggesting that enzymatic sugar-release assays performed using micronized biomass were not biased by the artefactual loss of plant cell wall components during micronization.

However, micronization can be seen as a mechanical pretreatment of biomass [17,18] as it enhances the enzymatic digestibility of substrates by increasing their specific surface (*i.e*., by reducing their size). Whereas ball milling (particle size < 0.05 mm) can also alter cellulose crystallinity [19,20] and hemicellulose structure and extractability [21], conventional (5-1 mm) and fine (≤ 0.1 mm) grinding do not seem to change the lignocellulosic structure. Since the aim of micronization in the present study was to allow substrate suspensions to be easily pipetted by the robot, but not to change the lignocellulosic structure, particle size reduction was limited to ~0.1 mm. For the same reason, centrifugal and impact millings were chosen because they are fast procedures and hence further reduced the probability of lignocellulosic structure alteration [12].

### Automated sugar-releasing assay

Four parameters were optimized to best fit with the requirements of the assay: amount of substrate, amount of enzyme, incubation time, and incubation temperature.

#### a) Substrate concentration

The working substrate concentration needs to meet conflicting requirements:

1) it should be high enough to allow reliable measurements of reducing sugars release, but

2) this release has to remain below microplate reader's saturation threshold so that additional release driven by fungal secretomes containing enzymatic activities complementing those of E508 can be detected;

3) the substrate suspensions must not clog the robot tips and need to enable reproducible dispensing into substrate-containing plates.

**Figure 5:**
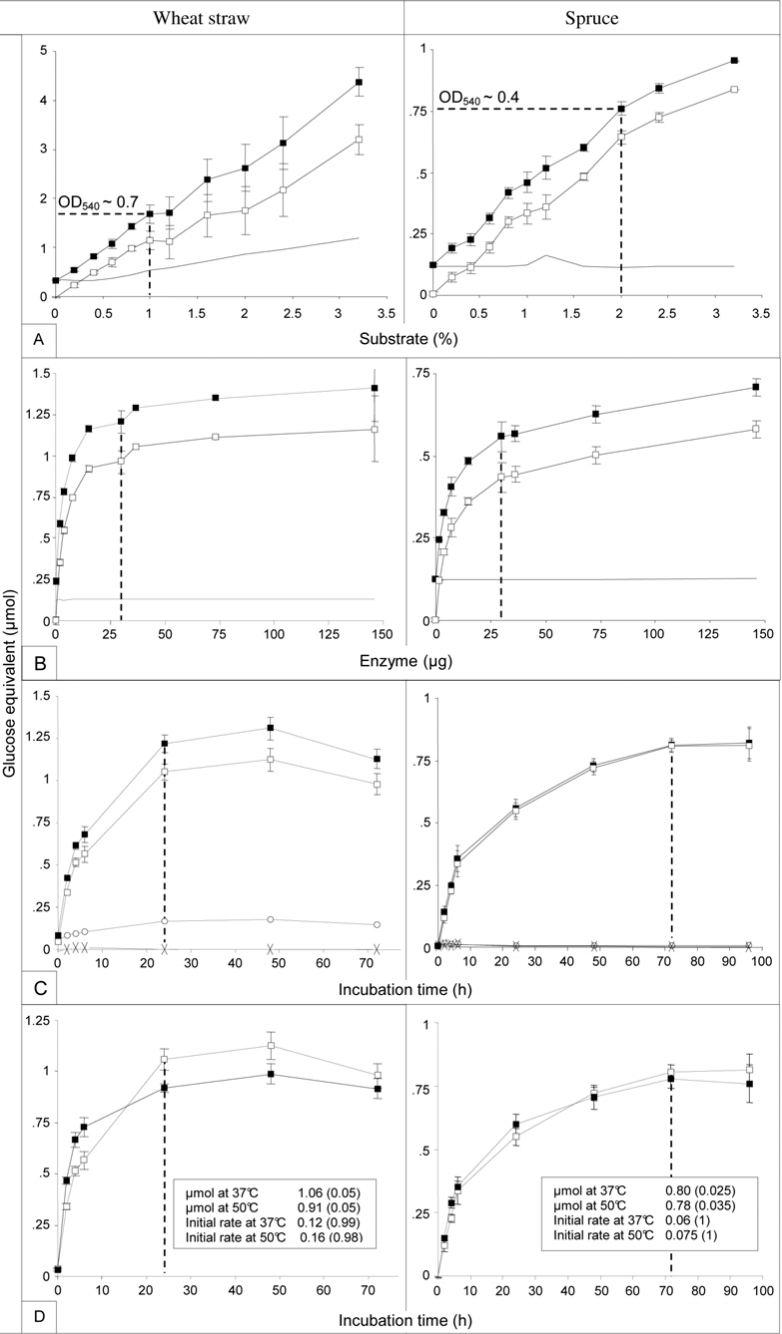
**Calibration of the sugar releasing experiment using micronized wheat-straw (left) or spruce (right)**. Except in D, black-square refers to raw data. Continuous line (A, B), open circle or cross (C) refer to background. White-square refers to raw data minus background. Standard deviations of triplicate experiments are indicated. Background curves were made of single measures. For each parameter, the value considered optimal and selected for subsequent experiments is flagged on the X axis by a vertical dotted line. A, Amount of reducing sugars released from micronized biomass as a function of substrate concentration. OD_540 _provided by the enzymatic digestion of 1% (wheat-straw) or 2% (spruce) substrate suspension are indicated on raw data curves. B, Amount of reducing sugars released from micronized biomass as a function of enzyme (E508) concentration. The continuous line is the "enzyme background". C, amount of reducing sugars released from micronized biomass as a function of incubation time. "Substrate background" (open circle), and "enzyme background" (cross) lines are reported. D, As for C except that only net sugar release curves are shown. Incubations were performed at 37°C (white square) or 50°C (black square). Inset: *μmol *of glucose equivalent are the values obtained at 24 h and 72 h incubation for wheat-straw and spruce, respectively (values in brackets are standard deviations); *initial rates *were calculated using data from 0 to 4 h time points, and were expressed as μmol glucose equivalent per hour (values in brackets are correlation factors).

Sugar release was tested with substrate concentrations ranging from 0 to 3.2% (w/v). The results are reported in Figure 5A. For the same substrate concentration, more reducing sugars were released from wheat-straw than from spruce, a result in line with two other studies: a) similar observations were reported by Zheng et al. [22] using two grasses and two woods; b) poplar had to be more stringently pre-treated than corn stover to achieve comparable glucan hydrolysis yields [23], which the authors attributed to different lignin and arabinoxylan compositions. Interestingly, a survey of the literature (Additional file 4) confirmed that spruce averages higher lignin content (27.5%) than wheat-straw (19.6%), suggesting that the results were at least partly due to different lignin contents.

For an unknown reason, in the absence of enzyme the amount of assayed sugars increased slightly with increasing wheat-straw concentrations but remained stable with increasing spruce concentrations,

In the presence of E508, the amount of sugars released from both substrates increased with substrate concentration. In subsequent experiments, we elected to use wheat-straw at 1% concentration and spruce at 2% as they satisfied criteria 1) to 3) above.

#### b) Enzyme concentration

The aim of this experiment was to define the amount of reference enzyme (E508) needed to satisfy criteria 1) and 2) described in the previous paragraph.

Sugar release from 1% (wheat-straw) or 2% (spruce) substrate suspensions was assayed in the presence of 0 to 146 μg E508 proteins. The results are reported in Figure 5B. Sugar-release curves were similar, but wheat-straw released more sugars than spruce, as already observed when increasing substrate concentrations were incubated in the presence of a constant amount of enzyme (Figure 5A). Sugar release from both substrates increased rapidly from 0 to 15 μg E508 proteins. From 30 to 146 μg, sugar release increased only moderately, suggesting that 30 μg approached the saturation point under our experimental conditions. Hence, 30 μg of E508 was selected for use with both substrates in subsequent experiments because i) it met the project's aim to screen for different enzymatic activities to those present in E508, and ii) using close-to-saturation E508 concentrations made the assay "blind" to E508-like activities in complementation experiments. However, the results in Figure 5B also indicated that our method could easily be adapted to other projects such as initial rates measurements (see below), in which case lower enzyme concentrations could be used with the additional benefit of lower experiment costs.

#### c) Incubation time

Durations of enzymatic digestion ranging from 0 to 72 h were tested using the substrate and enzyme concentrations defined above. The results are reported in Figure 5C. Although sugar release from wheat-straw peaked at 48 h, sugar release from spruce did not plateau, even at 72 h. This difference may be related to the different lignin contents evoked above. Therefore, an additional assay using spruce was performed at 96 h which confirmed that a plateau had already been reached at the previous time-point (72 h).

Incubation durations close to saturation (24 h for wheat-straw and 72 h for spruce) were chosen as standard incubation times for the reason given in the previous paragraph.

Under these conditions, 120 triplicate wheat-straw samples could be processed per day. This throughput can be doubled if the incubation time is reduced from 24 h to 5 h (such as for initial rates measurements).

#### d) Incubation temperature

Cellulolytic enzymes are generally used at ~50°C [24]. However, saccharification must be performed at lower temperatures when bioethanol is produced by simultaneous saccharification and fermentation [25].

Therefore, the release of reducing sugars from micronized wheat-straw or spruce was monitored as a function of incubation time at 37°C and 50°C. The results are reported in Figure 5D. Surprisingly, at the standard incubation time-point defined in the previous paragraph (72 h) comparable amounts of reducing sugars were released from spruce by E508 at both temperatures. Moreover, the amount of sugars released from wheat-straw was even higher at 37°C than at 50°C at the standard incubation time-point (24 h). The latter result could be explained by a higher initial rate at 50°C (0.16 μmol glucose equivalent per hour) than at 37°C (0.12 μmol glucose equivalent per hour, Figure 3D inset). Conversely, and in agreement with this hypothesis, slightly less dissimilar initial rates (0.075 and 0.06 μmol glucose equivalent per hour) were associated with comparable sugar releasing yields from spruce whatever the temperature and time-point considered.

Irrespective of the temperature, initial rates were unambiguously higher on wheat-straw (0.16 and 0.12 *vs *0.075 and 0.06 for spruce, Figure 5D inset), in line with the previous 3 experiments (Figure 5A-C) which showed that the same amount of enzyme (Figure 5B) released more sugars from the same substrate concentration (Figure 5A) in less time (Figure 5C) when wheat-straw was used. This result is also in agreement with Zheng et al. [22]. Although this was not our primary goal, the correlation factors in Figure 5D insets indicated that our method was also effective at measuring initial rates.

As previously observed (Figure 3C), for an unknown reason a lower amount of reducing sugars released from wheat-straw was assayed at time-point 72 h. This phenomenon was less pronounced at 50°C than at 37°C. It is noteworthy that using steam exploded chopped wheat-straw, Tabka et al. [15] observed an increased sugar release when the incubation temperature was shifted from 37°C to 50°C. Whether micronization and/or lack of pre-treatment could explain this discrepancy would be an interesting way of investigation.

On the basis of these results, 37°C was retained as reference temperature for the present study.

#### e) Summary

A summary of the whole assay including substrate plate filling is described in Figure 1.

A video illustrating this summary can be seen at the following URL: http://www.youtube.com/watch?v=NFg6TxjuMWU

### Validation of the automated assay using different enzyme sources

**Figure 6:**
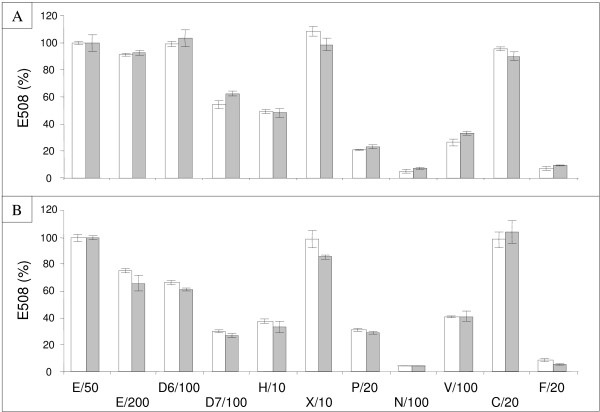
**Reducing-sugar release from micronized substrates by different enzymatic cocktails at 37°C and 50°C**. A, Micronized wheat-straw was incubated at 37°C (white histograms) or 50°C (gray histograms) in the presence of 15 μL of the indicated enzymes (X axis, see 'Methods' for details) for 24 h. B, As for A except that micronized spruce was used as substrate and incubation time was 72 h. In A and B, the results of three independent experiments, each performed in triplicate, are expressed as % of E/50 activity.

The assay was tested by comparing the amount of sugars released from micronized wheat-straw and spruce by the following commercially-available enzymes: E508 (E), Depol 686 L (D6), Depol 740 L (D7), Hemicellulase (H), Xylanase (X), Pectinex Ultra SP-L (P), Novozyme 188 (N), Viscozyme L (V), Celluclast 1.5 L (C), and Fungamyl 800 L (F). The enzymes are described in Additional file 3. They were used at dilutions that preliminary tests (Additional file 5) had indicated best fitted our criteria 1) and 2). These preliminary tests also defined 30 μg E508 (*i.e*. 25 μL of a 1/50 dilution (E/50)) as 100% sugar-releasing activity (reference activity). The results are reported in Figure 6A and 6B. An all-round look suggested a good capacity of the assay to discriminate among biomass-degrading activities ranging from almost 0 (N/100 - spruce) to more than 100% (X/10 - wheat-straw) with respect to the reference activity (E/50). In addition, the low standard deviations could be considered a global measure of the reproducibility of each step of the method (substrate micronization distribution and digestion, DNS assay, evaporation, microplate reading).

As already observed using E/50 only (Figure 5C), the results reported in Figure 6A and 6B confirmed with other enzymes that incubations performed at 37°C and 50°C provided similar results. Perhaps surprisingly, two substrates with very different structures showed rather similar digestion patterns in response to different enzymes, at least when compared to the reference activity. Among differences, spruce seemed to be more sensitive to E508 dilution (E/50 *vs *E/200) than wheat-straw, although the spruce concentration (2%) was twice that of wheat-straw (1%) in our assay. Wheat-straw was more sensitive than spruce to digestion by Depol 686 L and, to a lesser extent, Depol 740 and hemicellulase. Finally, both substrates exhibited roughly the same sensitivity to the other enzymes (Xylanase, Pectinex Ultra SP-L, Novozyme 188, Viscozyme L, Celluclast1.5 L, and Fungamyl 800 L).

Since the composition of these commercial enzymatic cocktails has not been precisely established only the results obtained with pure enzymatic activities could be further discussed. For instance, since Novozyme 188 (N/100) is a rich source of *A. niger *β-glucosidase, its low activity on micronized wheat-straw or spruce could therefore be considered a measure of the (low) amount of cellobiose generated by the micronization process. Also, the low efficiency of Fungamyl 800 L (*A. oryzae *α-amylase (F/20)) on micronized wheat-straw or spruce could be explained by the absence of starch in these substrates.

## Conclusions

We have set up an automated method which overcomes some of the problems typically encountered in biomass conversion research [26]: *i*) automating the entire process increased throughput; *ii*) the use of an automated plate-sealer made the whole process reliable by avoiding evaporation issues; *iii*) substrate micronization allowed uniform distribution into 96-well plates.

We currently use the automated assay described in the present paper in combination with our miniaturized fungal culture method [7] to screen the lignocellulolytic activity of hundreds of fungal secretomes from natural biodiversity (to be published elsewhere).

The method parameters (substrate and enzyme concentrations, incubation temperature and duration) were selected specifically for use in this trial, but could easily be modified for other purposes.

Provided they are micronized, other substrates such as natural substrates (miscanthus, switchgrass, poplar), industrial by-products (wheat bran, corn stover, rice straw, bagasse, brewer's spent grain, sugar beet pulp), or purified substrates (xylan, mannan, pectin, cellulose) could be processed with our method using different enzyme sources (bacterial lysates, purified enzymes alone or in combination). For instance, we successfully processed thermochemically pre-treated biomass (dried acid pre-treated steam exploded wheat-straw) in our method (DN, MC, JGB, unpublished results). Therefore, the method could be useful in other research areas such as feed and food (bread making), pollution remediation (reconstituted garbage), or basic science (enzymology).

## List of abbreviations

DNS: 3,5-dinitrosalicylic acid; rpm: round per minute; di: dispersion index; GLC: gas-liquid chromatography; Hz: Hertz.

## Competing interests

The authors declare that they have no competing interests.

## Authors' contributions

DN and MC set up the robotic platform and performed most of the experiments reported in this study. GGDdS and XR prepared, analyzed and provided the micronized wheat-straw and spruce. JGB and MA are managers for this part of the E-TRICEL project dealing with high-throughput characterization of fungal biodiversity. CB proposed the idea of a fully automated set-up, validated the first manual experiments, wrote the paper and managed the whole set-up.

## Supplementary Material

Additional file 1 **Supplementary Figure 1**. pictures of the two substrates used in this study at different steps of size reduction. Click here for file

Additional file 2 **Supplementary Figure 2**. this file provides information on the general organization of substrate-containing 96-well plates, and on well-to-well reproducibility of substrate slurry filling. Click here for file

Additional file 3 **Supplementary Figure 3**. this file provides a description of the commercially available enzymes used in this study and comprises 1) the fungus from which the enzymatic activity was recovered, 2) the company selling the enzyme, 3) the industrial use of the enzyme, 4) the main enzymatic activity, 5) the corresponding publication. Click here for file

Additional file 4 **Supplementary Figure 4**. this file provides a summary of published data on the assessment of lignin content in wheat-straw and spruce. Click here for file

Additional file 5 **Supplementary Figure 5**. this file provides the raw data of preliminary experiments performed using 2 enzyme dilutions so as to find out the dilution that best fitted with criteria 1 and 2 defined in the results section of the manuscript (automated sugar releasing assay: substrate concentration). Click here for file
