# Supplementary material for: Automated assay for screening the enzymatic release of reducing sugars from micronized biomass
Source: Microb Cell Fact. 2010 Jul 16;9:58. doi: 10.1186/1475-2859-9-58 (PMC2919459; doi:10.1186/1475-2859-9-58)
Supplement: Additional file 1 — Supplementary Figure 1. pictures of the two substrates used in this study at different steps of size reduction. [file 1475-2859-9-58-S1.PDF]

Process		Mincing	Grinding	Micronization	
Average size		0.5 - 5 cm	2 mm	< 0.3 mm	< 0.12 mm
Substrate	Wheat -straw	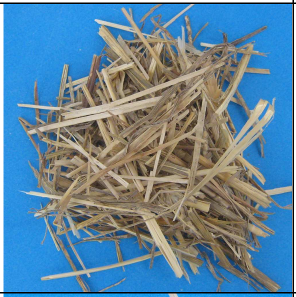	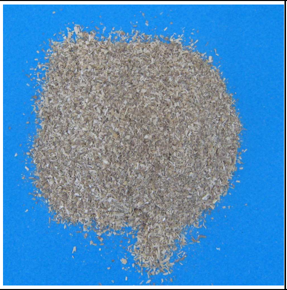	NA	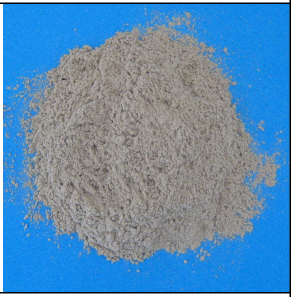
	Spruce	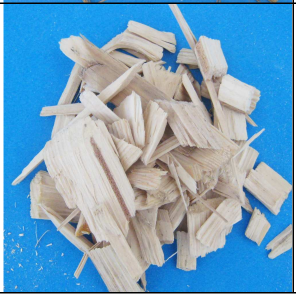	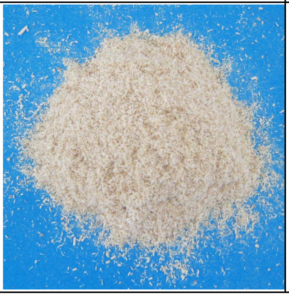	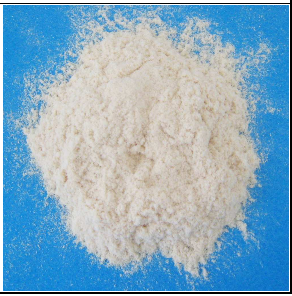	NA

Supplementary Figure 1. Pictures of the two substrates used in this study at different steps of size reduction. NA, not applicable.
