# Supplementary material for: Automated assay for screening the enzymatic release of reducing sugars from micronized biomass
Source: Microb Cell Fact. 2010 Jul 16;9:58. doi: 10.1186/1475-2859-9-58 (PMC2919459; doi:10.1186/1475-2859-9-58)
Supplement: Additional file 2 — Supplementary Figure 2. this file provides information on the general organization of substrate-containing 96-well plates, and on well-to-well reproducibility of substrate slurry filling. [file 1475-2859-9-58-S2.PDF]

A	1	2	3	4	5	6	7	8	9	10	11	12
A	0	0	E/200	D6/10	D7/10	H	X	P	N/10	V/10	C	F
B	2	0	E/200	D6/10	D7/10	H	X	P	N/10	V/10	C	F
C	4	0	E/200	D6/10	D7/10	H	X	P	N/10	V/10	C	F
D	6	0	E/200	D6/10	D7/10	H	X	P	N/10	V/10	C	F
E	8	E/50	E/200	D6/100	D7/100	H/10	X/10	P/20	N/100	V/100	C/20	F/20
F	10	E/50	E/200	D6/100	D7/100	H/10	X/10	P/20	N/100	V/100	C/20	F/20
G	15	E/50	E/200	D6/100	D7/100	H/10	X/10	P/20	N/100	V/100	C/20	F/20
H	20	E/50	E/200	D6/100	D7/100	H/10	X/10	P/20	N/100	V/100	C/20	F/20

B	Weight (mg)						Pipetting depth
Experiment	Empty plate	Plate after filling	Net weight		Mean	Standard deviation	
			Per plate	Per well			
1	49348	56241	6894	104	110	6	10 mm below the surface
2	48707	55927	7220	109			
3	48933	56715	7782	118			
4	48911	55953	7042	107			
5	49017	55906	6889	104	100	4	Z max
6	48736	55136	6400	97			
7	48955	55297	6342	96			
8	48658	55372	6714	102			

C							
1	40878	41197	319	106	104	2	10 mm below the surface
2	41197	41513	316	105			
3	41513	41823	310	103			
4	41823	42125	302	101			
5	42125	42431	306	102			
6	42431	42731	300	100			
7	42731	43046	315	105			
8	43046	43369	323	108			
9	43369	43679	310	103			
10	43679	43992	313	104			
11	40512	47844	7332	111,1	111	1	Z max
12	40521	47893	7372	111,7			
13	40617	47853	7236	109,6			
14	40521	47877	7356	111,5			
15	40583	47799	7216	109,3			
16	40637	47854	7217	109,3			
17	40667	47947	7280	110,3			
18	40556	47930	7374	111,7			

Supplementary Figure 2. Substrate-containing microplate.

A, **General organization of substrate-containing 96-well plate**. Well ID: wells were localized using column (1-12) / raw (A-H) binary system of standard 96-well microplates. All wells were filled with substrate except those in column 1 and in raw A and E (*i.e.* 66 wells (light grey) out of 96). Just before using the plate in an enzymatic assay, column 1 was extemporaneously filled with a reference glucose scale ranging from 0 (well A1) to 20 mM (well H1). Enzyme: 0, no enzyme; E/50, E508 (1/50 dilution); E/200, E508 (1/200 dilution); D6/10, Depol 686L (1/10 dilution); D6/100, Depol 686L (1/100 dilution); D7/10, Depol 740L (1/10 dilution); D7/100, Depol 740L (1/100 dilution); H, undiluted Hemicellulase; H/10, Hemicellulase (1/10 dilution); X, undiluted Xylanase; X/10, Xylanase (1/10 dilution); P, undiluted Pectinex Ultra SPL; P/20, Pectinex Ultra SPL (1/20 dilution); N/10, Novozyme 188 (1/10 dilution); N/100, Novozyme 188 (1/100 dilution); V/10, Viscozyme L (1/10 dilution); V/100, Viscozyme L (1/100 dilution); C, undiluted Celluclast 1.5 L; C/20, Celluclast 1.5 L (1/20 dilution); F, undiluted Fungamyl 800 L; F/20, Fungamyl 800 L (1/20 dilution). This example of enzyme dispensing was used in the experiment reported in Figure 6 and can be modified according to each experiment design.

B, **Plate-to-plate filling reproducibility of wheat straw suspension** (100 µl/well, ~100 mg) was evaluated when the uptake was performed at the top (*i.e.* 10 mm below the liquid surface, experiments 1 to 4) or at the bottom (*i.e.* just above the stirring magnet (Z max), experiments 5 to 8) of a 1% (w/v) powdered wheat-straw suspension under continuous stirring. In either case, 4 microplates (column 1) were weighted before (column 2) and after (column 3) filling. The net weight (difference between columns 2 and 3) was reported in column 4. For each plate, the net weight per well (column 5) was calculated by dividing the result in column 4 by the number of wells filled with substrate (*i.e.* 66, see A). The mean value (column 6) and standard deviation (column 7) were indicated for each pipetting depth (column 8).
C, **Plate-to-plate filling reproducibility of spruce suspension**. Same as B, except that *i*) a 2% (w/v) suspension was used, *ii*) uptake 10 mm below the surface was performed using 10 microlplates (experiments 1 to 10) and plates were weighted after 3 wells were filled (hence the net weight per plate around 300 mg (column 4)), *iii*) uptake at Z max was performed using 8 microplates (experiments 11 to 18).
