# Supplementary material for: Automated assay for screening the enzymatic release of reducing sugars from micronized biomass
Source: Microb Cell Fact. 2010 Jul 16;9:58. doi: 10.1186/1475-2859-9-58 (PMC2919459; doi:10.1186/1475-2859-9-58)
Supplement: Additional file 3 — Supplementary Figure 3. this file provides a description of the commercially available enzymes used in this study and comprises 1) the fungus from which the enzymatic activity was recovered, 2) the company selling the enzyme, 3) the industrial use of the enzyme, 4) the main enzymatic activity, 5) the corresponding publication. [file 1475-2859-9-58-S3.PDF]

Commercial name (letter used in Figure 6)	Organism	Provider	Main known activity	Industrial use	Reference
E508 (E)	*Trichoderma reesei CL847*	SAFISIS	Cellulase	Fermentable sugar release from cellulose	Durand *et al*. 1988. Enzyme Microb. Technol. 10, 341-346.
Depol 686L (D6)	*Trichoderma sp.*	Biocatalysts	Broad spectrum carbohydrases (β-glucanase and xylanase)	Viscosity reduction in brewing process	Faulds *et al*. 2008. J. Agric. Food Chem. 56, 7038–7043
Depol 740L (D7)	*Humicola sp.*		Ferulic acid esterase	Waste processing of natural products. Flavour production	
Hemicellulase [H2125] (H)	*Aspergillus niger*	Sigma [reference]	Xylanase, mananase and other activities	Animal feed. Paper bleaching Biopulping. Flour processing	
Xylanase [X3876] (X)	*Trichoderma viride*		Endo-1,4-β Xylanase	See hemicellulase	Wong *et al*. 1988. Microbiol. Rev. 52, 305.
Celluclast 1.5L [C2730-50] (C)	*Trichoderma reesei*		Crude extract of *T. reesei* cellulases.	Breakdown of cellulose into glucose, cellobiose, and higher glucose polymers	Reese and Mandels. 1980. Biotechnol. Bioeng. 22, 323- 335.
Pectinex Ultra SPL (P)	*Aspergillus aculeatus*	Novozyme	Pectinase	Fruit juice clearing	
Novozyme 188 (N)	*Aspergillus niger*		β-glucosidase	hydrolysis of steam-exploded biomass	
Viscozyme L (V)	*Aspergillus acuelatus*		Multi-enzyme complex containing a wide range of carbohydrases (arabanase, cellulase, β-glucanase, hemicellulase, xylanase…)	Increases ethanol production and lowers operating costs	Guan and Yao. 2008. Food Chemistry 106, 345-351.
Fungamyl 800 L (F)	*Aspergillus oryzae*		α-amylase	Increases starch breakdown and alcohol output.	Bernfeld, P. Meth. 1955 Enzymol. 1, 149.

Supplementary Figure 3. Description of the commercially available enzymes used in this study. “Organism” is the fungus from which the enzymatic activity was recovered. “Provider” is the name of the company selling the enzyme. “Main known activity” is the prominent enzymatic activity tested in the publication cited in the column “Reference”.
