# Supplementary material for: Automated assay for screening the enzymatic release of reducing sugars from micronized biomass
Source: Microb Cell Fact. 2010 Jul 16;9:58. doi: 10.1186/1475-2859-9-58 (PMC2919459; doi:10.1186/1475-2859-9-58)
Supplement: Additional file 4 — Supplementary Figure 4. this file provides a summary of published data on the assessment of lignin content in wheat-straw and spruce. [file 1475-2859-9-58-S4.PDF]

Substrate		Lignin (%)	Mean (%)	Reference
Wheat straw	*Triticum aestivum* c.v. Cadenza - internodes	19.3	19.62	Bertrand et al., 2009
	Triticum vulgare c.v. Horoshiri	19.0		Pan and Sano, 2005
	Triticum aestivum sp.	21.0		Lequart et al., 2000
	Triticum aestivum L. cv. Scipion	21.8		Durot et al., 2003
	Variety Riband	17.0		Xu et al., 2006
Spruce	Fresh chips	28.6	27.53	Zhu et al., 2009
	spruce	26.81		Schilling et al., 2009
	Picea abies	27.6		Assor et al., 2009
	Picea abies	27.11		Rousset et al., 2009

References.

Assor, C., Placet, V., Chabbert, B., Habrant, A., Lapierre, C., Pollet, B., Perre, P., 2009. Concomitant Changes in Viscoelastic Properties and Amorphous Polymers during the Hydrothermal Treatment of Hardwood and Softwood. *Journal of Agricultural and Food Chemistry*, 57, 6830-6837.
Bertrand, I., Prevot, M., Chabbert, B., 2009. Soil decomposition of wheat internodes of different maturity stages: Relative impact of the soluble and structural fractions. *Bioresource Technology*, 100, 155-163.
Durot, N., Gaudard, F., Kurek, B., 2003. The unmasking of lignin structures in wheat straw by alkali. *Phytochemistry*, 63, 617-623.
Lequart, C., Ruel, K., Lapierre, C., Pollet, B., Kurek, B., 2000. Abiotic and enzymatic degradation of wheat straw cell wall: a biochemical and ultrastructural investigation. *Journal of Biotechnology*, 80, 249-259.
Pan, X.J., Sano, Y., 2005. Fractionation of wheat straw by atmospheric acetic acid process. *Bioresource Technology*, 96, 1256-1263.
Rousset, P., Lapierre, C., Pollet, B., Quirino, W., Perre, P., 2009. Effect of severe thermal treatment on spruce and beech wood lignins. *Annals of Forest Science*, 66.
Schilling, J.S., Tewalt, J.P., Duncan, S.M., 2009. Synergy between pretreatment lignocellulose modifications and saccharification efficiency in two brown rot fungal systems. *Applied Microbiology and Biotechnology*, 84, 465-475.
Xu, F., Sun, J.X., Sun, R.C., Fowler, P., Baird, M.S., 2006. Comparative study of organosolv lignins from wheat straw. *Industrial Crops and Products*, 23, 180-193.
Zhu, J.Y., Wang, G.S., Pan, X.J., Gleisner, R., 2009. Specific surface to evaluate the efficiencies of milling and pretreatment of wood for enzymatic saccharification. *Chemical Engineering Science*, 64, 474-485.

Supplementary Figure 4. **Assessment of lignin content in wheat-straw and spruce: a summary of published data.**
