# Supplementary material for: Automated assay for screening the enzymatic release of reducing sugars from micronized biomass
Source: Microb Cell Fact. 2010 Jul 16;9:58. doi: 10.1186/1475-2859-9-58 (PMC2919459; doi:10.1186/1475-2859-9-58)
Supplement: Additional file 5 — Supplementary Figure 5. this file provides the raw data of preliminary experiments performed using 2 enzyme dilutions so as to find out the dilution that best fitted with criteria 1 and 2 defined in the results section of the manuscript (automated sugar releasing assay: substrate concentration). [file 1475-2859-9-58-S5.PDF]

Wheat-straw

Glucose scale		OD_{540}											
		No enzyme (0)	E508 (E/200)	Depol 686L (D6/10)	Depol 740L (D7/10)	hemicellulase (H)	Xylanase (X)	Pectinex (P)	Novozyme 188 (N/10)	Viscozyme (V/10)	Cellulclast (C)	Fungamyl (F)	
mM	OD_{540}	0,07	0,07	2,99	0,15	0,93	1,18	0,53	0,17	1,88	1,06	0,64	No substrate
		0,11	0,37	Overflow	0,43	0,88	0,90	0,52	0,28	1,20	1,14	1,20	Triplicates
0	0,07	0,15	0,47	Overflow	0,56	1,26	1,52	0,69	0,29	1,90	1,85	1,10	
2	0,10	0,12	0,32	2,92	0,30	0,94	1,05	0,50	0,12	1,63	0,68	0,52	
4<br>6	0,22<br>0,21	E508 (E/50)	E508 (E/200)	Depol 686L (D6/100)	Depol 740L (D7/100)	hemicellulase (H/10)	Xylanase (X/10)	Pectinex (P/20)	Novozyme 188 (N/100)	Viscozyme (V/100)	Celluclast (C/20)	Fungamyl (F/20)	
8	0,34	0,07	0,07	0,07	0,07	0,06	0,10	0,07	0,07	0,11	0,07	0,06	No substrate
10	0,51	0,70	0,58	0,73	0,39	0,38	0,85	0,24	0,16	0,40	0,70	0,21	Triplicates
15	0,75	0,61	0,54	0,58	0,40	0,39	0,56	0,23	0,16	0,34	0,53	0,18	
20	0,96	0,68	0,61	0,80	0,29	0,30	0,88	0,24	0,19	0,46	0,70	0,20	

Spruce

Glucose scale		OD_{540}											
		No enzyme (0)	E508 (E/200)	Depol 686L (D6/10)	Depol 740L (D7/10)	hemicellulase (H)	Xylanase (X)	Pectinex (P)	Novozyme 188 (N/10)	Viscozyme (V/10)	Cellulclast (C)	Fungamyl (F)	
mM	OD_{540}	0,07	0,10	Overflow	0,15	2,38	1,21	0,52	0,20	1,98	1,25	1,37	No substrate
		0,08	0,49	Overflow	0,43	2,75	1,92	1,03	0,32	2,54	1,88	1,32	Triplicates
0	0,08	0,08	0,52	Overflow	0,46	2,93	2,04	1,01	0,35	2,49	1,85	1,31	
2	0,12	0,07	0,50	Overflow	0,46	2,94	2,09	1,01	0,35	2,57	1,88	1,30	
4<br>6	0,24<br>0,37	E508 (E/50)	E508 (E/200)	Depol 686L (D6/100)	Depol 740L (D7/100)	hemicellulase (H/10)	Xylanase (X/10)	Pectinex (P/20)	Novozyme 188 (N/100)	Viscozyme (V/100)	Celluclast (C/20)	Fungamyl (F/20)	
8	0,44	0,07	0,07	0,08	0,07	0,08	0,13	0,08	0,08	0,23	0,07	0,10	No substrate
10	0,54	0,61	0,44	0,41	0,20	0,43	0,54	0,22	0,08	0,42	0,56	0,14	Triplicates
15	0,71	0,60	0,47	0,49	0,22	0,30	0,70	0,25	0,10	0,42	0,61	0,13	
20	0,99	0,59	0,49	0,44	0,22	0,30	0,62	0,24	0,10	0,43	0,59	0,14	

Supplementary Figure 5. The activity of the enzymes used in this study was assayed at two different dilutions so as to find out the dilution that best fitted with criteria 1) and 2) defined in the Results and Discussion section of the manuscript (automated sugar releasing assay: substrate concentration). The value indicated after the slash indicates the dilution. For instance: /10 means 1/10 diluted.
